# Using mHealth applications for self-care – An integrative review on perceptions among adults with type 1 diabetes

**DOI:** 10.1186/s12902-022-01039-x

**Published:** 2022-05-25

**Authors:** Divya Anna Stephen, Anna Nordin, Jan Nilsson, Mona Persenius

**Affiliations:** 1 Department of Health Sciences, Faculty for Health, Science And Technology, Karlstad University, Karlstad, Sweden; 2 Department of Health, Learning and Technology, Nursing and Medical Technology, Luleå University of Technology, Luleå, Sweden; 3 Faculty of Health and Social Sciences, Inland Norway University of Applied Sciences, Elverum, Norway

**Keywords:** Diabetes mellitus, mHealth, Mobile health, Mobile applications, Patient reported outcome measures, Self care, Self-management

## Abstract

**Background:**

Individually designed interventions delivered through mobile health applications (mHealth apps) may be able to effectively support diabetes self-care. Our aim was to review and synthesize available evidence in the literature regarding perception of adults with type 1 diabetes on the features of mHealth apps that help promote diabetes self-care, as well as facilitators and barriers to their use. An additional aim was to review literature on changes in patient reported outcome measures (PROMs) in the same population while using mHealth apps for diabetes self-care.

**Methods:**

Quantitative and qualitative studies focusing on adults aged 18 years and over with type 1 diabetes in any context were included. A systematic literature search using selected databases was conducted. Data was synthesised using narrative synthesis.

**Results:**

We found that features of mHealth apps designed to help promote and maintain diabetes self-care could be categorized into self-care data monitoring, app display, feedback & reminders, data entry, data sharing, and additional features. Factors affecting the use of mHealth apps reported in the literature were personal factors, app design or usability factors, privacy and safety factors, or socioeconomic factors. Quality of life and diabetes distress were the most commonly reported PROMs in the included studies.

**Conclusion:**

We are unable to reach a conclusive result due to the heterogeneity of the included studies as well as the limited number of studies reporting on these areas among adults with type 1 diabetes. We therefore recommend further large-scale studies looking into these areas that can ultimately improve mHealth app use in type 1 diabetes self-care.

**Systematic review registration:**

Prospero CRD42020157620.

**Supplementary Information:**

The online version contains supplementary material available at 10.1186/s12902-022-01039-x.

## Background

Diabetes can lead to a large economic burden being placed on individuals, the health-care system, and the wider global economy [1]. Acute complications caused by diabetes are a significant contributor to mortality, high costs, and poor quality of life [2]. People with type 1 diabetes with glycated hemoglobin (HbA1C) in the normal range face twice the risk of death from both cardiovascular and other complications when compared to the general population. This risk gradually increased with poor glycemic control [3]. Keeping blood sugar levels as close to the nondiabetic range as safely possible decreases the risk of complications caused by diabetes [4].

Self-care, an important aspect of diabetes care [5] is the process of maintaining health through health promoting practices and managing illness via self-care maintenance, self-care monitoring, and self-care management [6]. Self-care encompasses self-management, another widely used term in this context [7, 8]. However, self-care is sometimes considered as a component of self-management [9] or a synonym [8, 9]. In this study, we have chosen to use the term self-care. The seven diabetes self-care domains defined by the Association of Diabetes Care and Education Specialists (ADCES) are healthy eating, being active, taking medication, reducing risk, healthy coping, problem solving and monitoring. Here monitoring involves data monitoring of overall health including blood glucose levels, blood pressure, weight, cholesterol levels, heart health, sleep, mood, medications, and eye, kidney and foot health that contributes towards self-care by learning how different foods, activity and medication affect blood glucose [10]. Technological advances in blood glucose monitoring and the delivery of diabetes treatment have transformed diabetes self-care [11].

Digital diabetes technology, which includes mobile health applications (mHealth apps), can aid self-care and thereby improve the lives of people with diabetes [12]. Use of mHealth apps has been shown to improve diabetes outcomes [13–16]. Nevertheless, among the numerous mHealth apps for diabetes management currently available on the market, many lack vital features such as automated data entry (via wireless or bluetooth etc.) or reminders to check blood glucose, and do not comprehensively address all the self-care needs of people with diabetes [17]. Research has also indicated that people do not adhere to mHealth app use with reports of participant engagement decreasing progressively over time [18], with long-term use [19]. Reported barriers to use were lack of awareness of existing mHealth apps and features, technical literacy barriers, lack of recommendation to use by health care providers [20, 21], personal factors such as refusing to take accountability, lack of motivation [20], unfriendly app designs, and cost [21]. Thus, there exists a mismatch between the intended use of mHealth apps and people’s real-world experiences [22].

We therefore thought that examining people’s perspectives on mHealth app features that help adhere to diabetes self-care, barriers to their use and patient reported outcome measures (PROMs) might be of benefit. Thus, our aim was to review and synthesize available evidence in the literature regarding perception of adults with type 1 diabetes on the features of mHealth apps that help promote diabetes self-care, as well as facilitators and barriers to their use. An additional aim was to review literature on changes in patient reported outcome measures in the same population while using mobile health applications for diabetes self-care.

## Methods

### Design

We performed an integrative review to explore the following from a global perspective; 1) Which mHealth app features help promote and maintain diabetes self-care as experienced by people with type 1 diabetes? 2) What are the factors affecting the use of mHealth apps in diabetes self-care? 3) How do mHealth apps for diabetes self-care affect PROMs? We chose this design as we had decided to include diverse studies to answer our research questions [23]. This review was conducted according to the Cochrane handbook [24]. Reporting of this study has been carried out according to PRISMA guidelines [25]. The study protocol (CRD42020157620) was registered with the International Prospective Register of Systematic Reviews (PROSPERO). Table 1 below depicts the eligibility criteria for the inclusion and exclusion of articles in this review.

**Table 1:** Inclusion and exclusion criteria

Inclusion Criteria	Exclusion criteria
1. Studies with adults (≥ 18 years) 2. Type 1 diabetes 3. Studies focusing on mHealth apps^a^ 4. Studies carried out in any country and settings (such as primary care, outpatient or community settings) 5. Published in peer reviewed scientific journals 6. Published in English	1. Studies focusing on mHealth apps for diabetes prevention or pre-diabetes or type 2 diabetes 2. Studies with pregnant women or children with diabetes as the study population 3. mHealth apps addressing single self-care domains like physical activity, diet management, educational aspects or psychosocial aspects alone 4. Studies focusing on reviewing apps in application stores, review studies, technical design, or technical evaluation of mHealth apps (irrelevant study designs) 5. Studies conducted before 2010 6. Studies with software applications that are solely web-based and can only be accessed through an internet browser application in the mobile device 7. Studies where the outcomes of interest were not measured or reported or were irrelevant (common irrelevant outcomes were for example HbA1C, time or percentage time in target blood sugar range, quality adjusted life years, economic outcomes etc.)

^a^ mHealth apps here are defined as software applications run on mobile devices (smartphone, tablet, or smartwatch etc.) and operated by people with type 1 diabetes for self-monitoring of parameters, specifically Blood glucose and/or Insulin dose or Insulin bolus calculation, were included. Additional monitoring features such as tracking diet, exercise, mood, graphical trends, alerts to deviant values, diabetes education, and feedback from health care professionals are desirable but not necessary for inclusion. Devices like connected automated insulin delivery systems, app-based therapeutic decision support, flash or continuous glucose monitors etc. when accompanied by the use of a software applications run on a mobile or handheld devices was considered for inclusion

### Outcomes

Outcome measures were focused on people’s perspective on usage of mHealth apps. Here specifically they were features of mHealth apps promoting diabetes self-care as experienced by people with type 1 diabetes, factors affecting mHealth apps use in diabetes self-care and changes in PROMs (e.g. diabetes empowerment level, fear of hypoglycaemia, perception of problem areas in diabetes, and quality of life), in studies on mHealth apps for diabetes self-care.

### Information sources and search strategy

We performed a systematic literature search using PubMed, CINAHL, Web of Science, Scopus, PsycINFO, and IEEExplore. Trial registries such as WHO ICTRP as well as clinicaltrials.gov were searched initially to find any trials that might have been missed in searches. Pilot searches were performed using identified keywords (Table 2) and a librarian helped revise the search strategy. The search terms were kept broad in order to capture all studies reporting mHealth apps in diabetes self- care. The search strategies were tailored to each database (Table S1). Initial literature searches in all databases were conducted in March 2020 and updated in June 2021.

**Table 2:** Search terms

Population	diabetes mellitus OR “non insulin dependant diabetes mellitus” OR “type 2 diabetes mellitus” OR T2DM OR “insulin dependant diabetes mellitus” OR “type 1 diabetes mellitus” OR T1DM
Intervention	"medical informatics" OR "health informatics" OR "medical informatics applications" OR "digital health" OR "mobile application*" OR "mobile app*" OR "mobile medical application*" OR telemedicine OR mhealth OR m-Health OR "mobile health" OR telehealth OR telemonitor* OR tele-monitor* OR ehealth OR e-health OR smartphone* OR "nursing informatics" OR telenursing
Outcome	“self manage*” OR “self care” OR “self monitor*” OR “self evaluat*” OR “self assess*” OR “blood glucose self monitoring”

### Data selection and extraction

The reference management software EndNote™ 20 was used to organize references retrieved from various databases and to remove duplicate studies, which were also removed manually. The decisions made by the reviewers during screening stages were documented using Rayyan QCRI software [26]. Paired reviewers independently assessed each stage of the review process, from study selection to data synthesis and analysis. Disagreements were discussed by all four authors until consensus was reached. In line with Cochrane guidelines, we were over-inclusive in the title and abstract screening stage including all studies on mHealth app use with a broad outcome focusing on diabetes self-care (for e.g. studies with HbA1C, time in normal blood sugar range etc. as outcome). It was in the full text screening stage, we focused on looking for studies with our specific outcomes of interest. This helped prevent missing any eligible study where our specific outcomes of interest were listed as a secondary outcome in full text alone and not in abstract or title. The references given in studies meeting the inclusion criteria were examined for relevant articles that could have been missed in the initial search, however, this revealed no new eligible studies.

The following characteristics were extracted in individual studies: Publication details, methodology, participant characteristics, intervention/exposure, control/comparison, outcomes of interest focusing on people’s experience (mHealth app features, factors affecting use and PROMs) and additional relevant information such as ethical clearance, study protocol registration, conflict of interest or bias. Key findings from each included study are summarized and presented as a matrix (Table 3).

**Table 3:** Characteristics of included studies

Study & Year	Country	Aim	Study design	Population & setting	Intervention/expo-sure	Developer/ manufact-urer	Outcome extracted^a^
Boyle, L., et al. (2017) [27]	New Zealand	To establish whether people with diabetes use apps to assist with diabetes self-management and which features are useful or desirable	Cross-sectional design; web based survey	Adults with diabetes^b^ recruited from a secondary care diabetes outpatient clinic; N (with T1D) = 105	Not applicable	Not applicable	Features, factors affecting use
Di Bartolo, P., et al. (2017) [28]	Italy	To compare iBGStar + DMApp with a traditional glucose meter in type 1 diabetes adolescents/ young adults	Randomized controlled trial	Type 1 diabetes subjects aged 14–24 years, treated with insulin, HbA1c ≥ 8.0%, and poor SMBG compliance recruited from 21 diabetes clinics; N (Adults) = 81	iBGStar™ glucose meter (MDR^$^ class 2) + iBGStar™ Diabetes Manager Application (MDR^$^ class 1) installed on the iPod touch or iPhone OS was used for 12 months	Sanofi Agamatrix Inc	PROM (AADQoL 19)
Drion, I., et al. (2015) [29]	Netherlands	To investigate the effects of the DBEES mobile phone diary application, on quality of life for patients with T1DM along with diabetes-related distress, HbA1c, SMBG, and usability of the diabetes application	Randomised controlled trial	Adults aged ≥ 18 years with T1DM, treated with insulin and own a smartphone recruited from a diabetes outpatient clinic; *N* = 63	The DBEES smartphone application and a linked personal web portal to enter diabetes-related self-care data used for 3 months	Freshware, Poland	PROMs (RAND 36, PAID)
Feuerstein-Simon, C., et al. (2018) [30]	USA	To examine the real-world use of a smartphone app, which receives meter readings and logs hypoglycemic symptoms, causes, and treatments to reduce hypoglycemia	Quasi-experimental design; Pilot study	Adults aged ≥ 21 years with T1D & current use of a smartphone recruited at the Joslin Diabetes Centre; *N* = 22	Joslin HypoMap™ app powered by Glooko to track hypoglycemic events & symptoms used for 12 weeks	Dr Howard Wolpert and powered by Glooko	PROM (Clarke’s survey)
Jeon, E., & Park, H. A. (2019) [31]	South Korea	To evaluate a diabetes self-care app by measuring differences in diabetes self-care factors between before and after using the app with the Information-Motivation-Behavioral skills model of Diabetes Self-Care	Quasi-experimental design	Adults aged ≥ 19 years with diabetes^b^ and own an android smartphone recruited through self-help websites for patients with diabetes; N (T1D) = 8	A research group developed diabetes self-care application used for 4 weeks	Author developed	PROMs (D-SMART, DFBC)
Kirwan, M., et al. (2013) [32]	Australia	To examine the effectiveness of a freely available smartphone application combined with text-message feedback to improve glycemic control and other diabetes-related outcomes in adult patients with type 1 diabetes	Randomized controlled trial	Adults aged 18–65 years with T1DM > 6 months, HbA1c > 7.5%, treated with insulin and own an iPhone recruited nationwide online; *N* = 72	Glucose Buddy, a free diabetes self-management iPhone application used for 9 months and Certified diabetes educator weekly review of data entered for 6 months	Skyhealth LLC	PROMs (DQoL, DES-SF, SDSCA)
Knight, B. A., et al. (2016) [33]	Australia	The aim of this study was to obtain user feedback on the usability of the RapidCalc app in adults with T1DM towards identifying user preferences and further development of this application	Qualitative focus group interview with thematic analysis	Adults aged 18–65 years with T1DM who were recent graduates of a flexible insulin management education program with HbA1c 7–10%; *N* = 7	A locally developed RapidCalc mobile phone app for diabetes self-care and specifically for flexible insulin management used for 1 month	Locally developed and acquired by A. Menarini Diagnostics	Features, factors affecting use
Mora, P., et al. (2017) [34]	USA	To assess the impact of using the Accu-Chek Connect diabetes management system on treatment satisfaction, diabetes distress, and glycemic control in adults with type 1 diabetes and insulin-treated type 2 diabetes	Quasi-experimental design	Adults aged ≥ 18 years with poorly controlled T1D experienced with Smartphone use recruited from primary care practices and diabetes specialty practices; N = 10	The Accu-Chek- Connect diabetes management system (MDR^$^ class 2) used for 6 months	Roche diabetes care	PROMs (DDS, DTSQ)
Ritholz, M. D., et al. (2019) [35]	USA	To explore qualitatively PWDs’ experiences using the integrated Sugar Sleuth technology to better understand how their experiences affected their diabetes self-management	Qualitative descriptive design, with thematic analysis	Adults aged 25–75 years with T1D ≥ 1 year, treated with insulin and HbA1c 7.5% -9.5% recruited at a diabetes specialty center; *N* = 10	The Sugar Sleuth system consisting of FreeStyle Libre, a wearable glucose sensor, and a mobile phone app used for 14 weeks	Abbott diabetes care	Features, factors affecting use
Skrøvseth, S. O., et al., (2012) [36]	Norway	To explore how self-gathered data could help users improve their blood glucose management	Quasi-experimental design	Adults with T1D attending a university Hospital; *N* = 30	The Few Touch Application (FTA)	Norwegian Centre for Integrated Care and Telemedicine (NST)	Factors affecting use
Tack, C., et al. (2018) [37]	Netherlands	To evaluate a prototype integrated mobile phone diabetes app in people with type 1 diabetes	Quasi-experimental design	Adults 18–65 years, with T1D ≥ 2 years, stable HbA1c 7%-10%, on variable bolus insulin dose and using a smartphone recruited at the outpatient clinics of a university hospital; *N* = 20	A prototype of an integrated mobile diabetes app used for 6 weeks	The Radboud University Medical Center, Royal Philips-the Netherlands & Salesforce (USA)	Features, factors affecting use, PROMs (CIDS, PAID, HFS)
Trawley, S., et al. (2017) [38]	Australia	To investigate the frequency of diabetes specific app use among a sample of adults in Australia with Type 1 or Type 2 Diabetes	Cross sectional survey	Adults aged 18–75 years with T1D recruited via a national level web-based survey; N (T1D) = 795	Not applicable	Not applicable	Features, factors affecting use
Zahed, K., et al. (2020) [39]	USA	To understand diabetic patients’ perceptions of hypoglycemic tremors, as well as their user experiences with technology to manage diabetes, and expectations from a self-management tool	Cross sectional survey	Adults aged ≥ 18 years with T1DM recruited via a national level web-based survey; *N* = 212	Not applicable	Not applicable	Features
Årsand, E., et al. (2015) [40]	Norway & Czech republic	To explore the interoperability and usability of a wearable computing device in conjunction with a developed smartphone application, and to evaluate its use in diabetes self-management	Quasi-experimental design; Usability study	Adults with type 1 diabetes recruited from an earlier NST project and affiliates from Motol University Hospital, Prague; *N* = 6	A Pebble smartwatch diabetes diary app for self-care data entry & tracking used for 2 weeks	Faculty of Biomedical Engineering, Czech technical University in Prague & NST	Features, factors affecting use

^a^ Outcomes extracted in relation to patient perspective, ^b^ Only results for T1D participants included in this study

*AADQoL* Audit of diabetes dependent quality of life, *BP* Blood pressure, *CIDS* Confidence in diabetes self-care, *DDS* Diabetes distress scale, *DES-SF* Diabetes empowerment scale short form, *DFBC* Diabetes self-management assessment report tool, *DQoL* Diabetes quality of life, *D-SMART* Diabetes self-management assessment report tool, *DTSQ* Diabetes treatment satisfaction questionnaire, *HCP* Health care professional, *HFS* Hypoglycemia fear survey, *MDR*^*$*^ Medical device regulation class as per US food and drugs administration, *NST* Norwegian Centre for Integrated Care and Telemedicine, *OPD* Outpatient departments, *PAID* Problem areas in diabetes, *RAND* 36 health-related quality of life, *RCT* Randomized control trial, *SDSCA* Summary of diabetes self-care activity, *SMBG* Self-monitoring of blood glucose, *T1DM* type 1 diabetes mellitus

### Quality appraisal

The following quality assessment tools were used in this study: Joanna Briggs institute quality appraisal tools for randomized control trials, quasi-experimental designs, cross sectional surveys, and qualitative designs. Two authors independently performed quality appraisals of all included studies (Table S2). Discrepancies were discussed among all four authors until resolved. We classified studies satisfying more than 70% of the appraisal tool criteria as high quality, 50–69% as moderate quality, and less than 50% as low quality. We arrived at these percentages by calculating the percentage ratio of questions in each quality appraisal form that were answered “yes” to the total number of questions. Assessment criteria answered as “unclear” were treated as a “no” i.e. not meeting the criteria. Pilot trials in our study were not categorized as high, moderate, or low quality. We have also chosen to include ethical approval status and conflict of interest in the quality appraisal table (Table S2), although we did not use these two criteria to assign a quality rating.

### Data synthesis

We performed a narrative synthesis of extracted data due to the heterogeneity in the included studies. The stages in this method are developing a theory of how the intervention works, why and for whom, developing a preliminary synthesis of findings of included studies, exploring relationships in the data, and assessing the robustness of the synthesis [41].

## Results

**Fig. 1:**
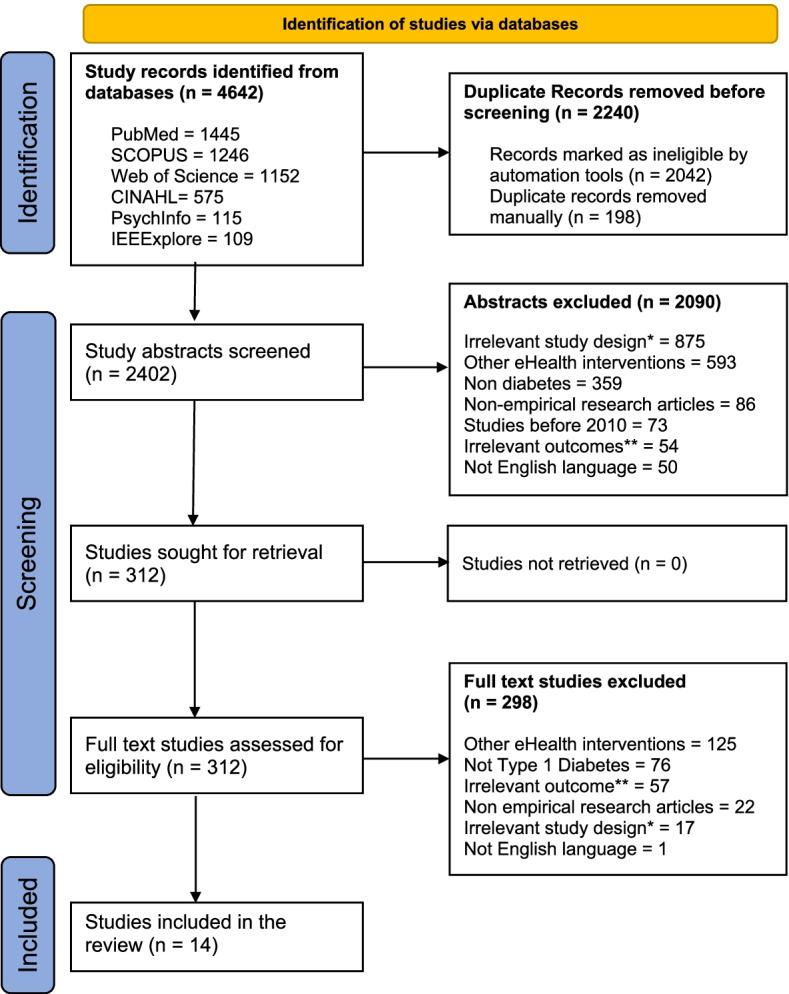
PRISMA 2020 flow diagram adapted from Page MJ et al. [25]. Refer to *point 4 and **point 7 under exclusion criteria in Table 1

A total of 4642 studies were retrieved from database searches. After removing duplicates, we screened the titles and abstracts of 2402 studies from which 312 studies were selected for full text screening. We excluded 298 studies during this stage due to reasons such as other eHealth interventions, population not having type 1 diabetes, irrelevant outcome, non-empirical research articles, irrelevant study design, and written in a language other than English. This resulted in 14 studies being included in the narrative synthesis (see Fig. 1 for PRISMA flowchart).

The eligible studies were published during the period 2012 to 2020. Five studies were from Europe, four were from North America, four were from Australia/New Zealand, and one was from Asia. The included studies had a range of study designs including randomised controlled trials (*n* = 3), quasi-experimental design (*n* = 6), cross sectional survey design (*n* = 3), and qualitative (*n* = 2). Of the quasi-experimental design studies, four were pilot trials. Seven of the included studies had mHealth app demonstrations before beginning the mHealth intervention [28, 30, 31, 33, 34, 37, 40] whereas the others did not report any demonstrations. In addition, these educational demonstrations varied from face-to-face demonstrations to distributing user guides, showing tutorial videos or all of these. Only three of the included studies reported offering technical support during the trial period [28, 37, 40]. The quality of the studies varied from high to moderate for the ten included studies that were not pilot trials. Study characteristics have been summarised in Table 3. The results of mHealth app features and factors affecting use have been arranged in the descending order of vote count.

### mHealth app features that help promote and maintain diabetes self-care

Based on our literature review, we have categorized features that promote and maintain diabetes self-care from the perspective of people with type 1 diabetes, into self-care data monitoring features, app display features, feedback & reminders, data entry features, data sharing features, and additional features (Table S3). The self-care data monitoring was the most common feature mentioned in the literature followed by app display, feedback and reminders, data entry, and data sharing features.

#### Self-care data monitoring

##### Diet

Food database connectivity or a food diary was a self-care data monitoring feature that was either favored for use by people using apps [38] or named as a desired feature [27, 33, 39, 40]. This feature was appreciated as it can assist with carbohydrate tracking [27, 33]. In another study, a carbohydrate counter was found to be the most commonly used feature among people with type 1 diabetes [38]. Another desired feature relating to diet was a shortcut option to ‘register’ favorite foods, as this enabled easy access [33].

##### Insulin log & bolus calculator

An insulin log and insulin bolus calculator was a feature that was either frequently used [27] or named as a desired feature by people with type 1 diabetes [27, 39]. The app features that study participants named as useful included the ability to customize time settings for insulin algorithms, the reverse (carbohydrate) calculator that helps prevent/manage over-treatment of hypoglycemia, and the insulin bolus adjustment feature for alcohol intake that enables reflection on the effect of alcohol on blood glucose levels [33]. Participants did comment, however, that the insulin bolus suggestions could be more specific and tailored for different activities e.g. the app could have a sports mode function [37].

##### Blood glucose

Among people with type 1 diabetes, recording blood glucose levels was a commonly used [27, 38] and sought-after feature [39]. The way blood glucose was recorded is not reported in two of the studies [27, 38] whereas the other report the use of either blood glucose monitors or continuous glucose monitors by participants [39]. A log for abnormal blood sugar was also sought after [39].

##### Physical activity

Features that could be used to track physical activity were also commonly used among people with type 1 diabetes [38]. Study participants appreciated being able to automatically import physical activities from their smartwatch to their smartphone app [40].

#### App display features

People wanted personalized screen displays allowing the removal (or cloaking) of features to simplify the user interface [33]. The graphical display of blood glucose (trend) was a desired feature among people with type 1 diabetes [39] and was also found to promote app use [37]. However, study participants wanted a more advanced method of graphically presenting results [37]. Graphs in the mHealth app led to less worrying about hypo- or hyperglycemia [35]. In one study that used a combined smartwatch & smartphone app, participants wanted to be able to view blood glucose graphs on their smartwatch and their most recent measurement values [40].

#### Feedback & reminders

Reminders were a desired feature [39], in particular reminders to check blood glucose were appreciated [27, 40]. One study found that constant and immediate feedback increased understanding and management of glucose variability [35].

#### Data entry features

Study participants wanted automated data transmission from their blood glucose meter [33, 37] and insulin pumps [37] via wireless or bluetooth connectivity to their smartphone app. They appreciated being able to register data quickly and preferred it to be in the order blood glucose, insulin, then carbohydrates [40]. They also wanted to be able to retrospectively edit self-monitoring data and be able to choose from a predetermined list of events that may be linked to variations in blood glucose [33]. People wanted to be able to record pertinent events (illness, stress etc.) [33, 40] using free text or an additional information option [33].

#### Data sharing feature

Study participants were looking for features that allowed for the easy export of their self-care data from their smartphone to a cloud solution or a connection with their electronic personal health record [37]. Some required additional web-based storage primarily for their blood glucose diary [33].

#### Additional features

Medication logs & educational content were other desired features [39], however these were reported less in the included literature.

### Factors affecting the use of mHealth apps in type 1 diabetes self-care

We found that factors affecting the use of mHealth apps reported in the included studies can be broadly categorized as personal factors, app design or usability related factors, privacy & safety factors, and socioeconomic factors. The most commonly reported factor was personal factors followed by app design or usability related factors.

#### Personal factors

In one study, the lowest percentage of app users was found in the age group 56 years or above [38]. However, another study found that increased mHealth app usage has been positively correlated with increasing age [36]. Some study participants found it convenient to enter data onto a smart phone, claiming that it enables “better record keeping” [33]. Some personal factors reported that could hinder use included a belief that apps would not help with their diabetes management or not having been able to find any good apps yet [38]. Some study participants were reluctant to use mHealth apps as they were perceived to demand too much information or that they demanded information at inconvenient times such as during an episode of low blood sugar [35].

#### App design/usability related factors

Design and technical concerns such as the complexity of the app or that it was not user friendly [37] can affect mHealth app use. Difficulty in converting macro data to raw data [33], inability to retrospectively input data [33, 37], or to correct errors in data [33], and use of outdated hardware [36] were some of the issues reported regarding app design flaws that hindered use.

#### Privacy & safety factors

mHealth app use can be affected by concerns about the validity of resources such as when insulin bolus calculator predications do not match a person’s own calculations or when a food database is not comprehensive enough [37].

#### Socioeconomic factors

One study reported that mHealth app interventions that did not cater to diverse socioeconomic backgrounds including those who have access to fewer resources [35] would lead to lower rates of use.

### Impact of mHealth apps for diabetes self-care on patient reported outcome measures

We found a wide range of PROMs reported in relation to mHealth app use for self-care among type 1 diabetes. Quality of life and diabetes distress were the most commonly used PROMs in the included studies. Among the PROMs, diabetes self-care social motivation [31], and diabetes treatment satisfaction [34] were found to significantly improve with mHealth app use. In addition, one study reported a statistically significant decrease in mean diabetes-related emotional problems, which is one of the subscales of the instrument measuring diabetes distress [37]. However, mHealth app use was not found to significantly improve quality of life [28, 29, 32], diabetes self-efficacy [32, 37], hypoglycemia fear [37], diabetes self-care activity [31, 32], and diabetes distress as a whole [29, 34, 37] in any of the included studies. The various PROM scales used in each study is indicated in Table 3.

## Discussion

In conducting this study, we sought to identify mHealth app features perceived by persons with type 1 diabetes to promote and help maintain diabetes self-care, factors that affect the use of mHealth apps, and the impact of mHealth apps on PROMs. Self-care data monitoring, which has been identified in this study as a frequently used or desired feature in mHealth apps, is also one of the key concepts in the middle-range theory of self-care of chronic illness. The Self-care data monitoring feature along with app display, feedback and reminders, data entry, and data sharing features help the underlying processes of self-care i.e. decision making and reflection that contribute to better self-care maintenance and management [6]. Self-care data monitoring feature also corresponds to ADCES7 self-care domains [10] of healthy eating, being active, monitoring, and taking medication, whereas other features reported important in the included articles corresponds to domain of reducing risk. The domains healthy coping and problem solving were not found to be desired features in our study, which is in line with findings from another review [42]. This may be due to a lack of incorporation of such features in the interventions reported here even though WHO recommends a holistic approach to self-care interventions, including those designed for people with diabetes [43].

We were unable to find similar reviews looking at mHealth app features from the perspectives of people with type 1 diabetes for comparison although studies listing or mapping features of available mHealth apps do exist [44–46]. Similar to our findings, one review on mHealth app intervention in general found that app display features such as personalisation, interface etc. do increase user engagement [47]. Only seven of the 14 studies included here reported on features, which indicates a need for more research in this area.

Similar to our findings on factors affecting mHealth app use, other systematic reviews have reported app design factors [22, 48], including privacy, and personal factors including socioeconomic factors to impact mHealth app use [49]. Socioeconomic factors may have a major affect on mHealth app use considering the fact that using these apps requires internet access and app subscriptions. However, this was only mentioned in one study included in our review. This may be due to the fact that studies meeting our inclusion criteria were only from high-income economies [50] despite us taking a global perspective. Similarly, privacy and safety factors were mentioned only by one included study as factors that can affect mHealth app use, while two studies reported that participants prefer having data sharing features incorporated in the app. Privacy concerns during the process of information collection and transmission over the internet may affect mHealth app use [49] and this is an area worth further exploration in future studies.

Among the included studies, only seven studies report on factors preventing mHealth app use. This data cannot be considered conclusive due to the limited number of studies included. Therefore, more large-scale studies exploring factors affecting mHealth app use are needed. Such data is helpful in identifying potential motivators and inhibitors to mHealth app use which in turn can help healthcare professionals in supporting patients’ mHealth app use as well as app developers to make their products more user friendly.

PROMs are a standardized way of quantifying people’s perspectives on the impact of illness and treatment to help assess the delivery of appropriate patient care [51]. We chose to look into PROMs instead of commonly studied measures such as HbA1C, time spent in normal blood sugar range, etc., because they help to capture the preferences of patients [52] which is an important aspect of value based healthcare [53, 54] and is therefore, important in the evaluation of the role played by mHealth apps in diabetes self-care. Most PROMs reported in the included studies did not significantly improve with mHealth app use. A probable reason for this may be that the included studies were trials completed in limited periods of app usage ranging from two weeks to one year, which may not have been long enough to capture statistically significant changes in PROMs. PROMs are only of value if they are well designed and based on a sound conceptual model and have scientific rigour [51]. A review of the methodological quality of PROM-based questionnaires used among people with type 2 diabetes showed that only 43 out of 238 identified PROMs met COSMIN guidelines indicating suitability to use [55]. The International Consortium for Health Outcomes Measurement (ICHOM) recommends PROMs that matter most to persons with Diabetes [56]. Among the three recommended PROMs only problem areas in diabetes (PAID) has been reported in the included studies whereas WHO-5 (well-being index) and Patient Health Questionnaire (PHQ-9) are not reported.

Our findings on PROMs are in line with reviews where mHealth app use did not show improvements in quality of life or self-care behaviors among people with type 1 [57] and people with type 2 diabetes [58], diabetes distress among people with type 2 diabetes [58], and diabetes-related self-efficacy among people with type 1 diabetes [57]. In contrast to our findings, diabetes treatment satisfaction showed no improvement in another study [57]. Also contrary to our results, another meta-analysis of people with type 2 diabetes showed significant improvement in self-efficacy, self-care activities, and health related quality of life among the mHealth app intervention group [59]. Of the seven included studies reporting on PROMs, three were pilot feasibility trials. More well-designed controlled trials on PROMs in this area will help healthcare professionals and organisations identify mHealth apps that can improve self-care among people with type 1 diabetes.

### Methodological consideration

Our review shows that peer reviewed scientific journal articles reporting on patient perspectives on mHealth app usage for self-care like desirable features, factors affecting their use as well as changes in PROMs are scarce, among people with type 1 diabetes. This was indicated by the fact that of the 2402 title and abstracts screened only 14 studies qualified to be included in our paper. It was also notable that we couldnot find many long-term follow-up studies reporting on PROMs in relation to mHealth app use. This is inspite of employing a search strategy that was systematic and comprehensive yet still broad, in order to capture all available literature that could answer our research question. We even chose to include keywords like “type 2 diabetes” in order to not miss articles with subgroup results for both type 1 and type 2. However, there is the risk that we may have missed studies if they did not use keywords such as self-care or self-management. Additionally, we may have missed studies published in languages other than english. To ensure individual study quality we included only published peer reviewed artcicles. The involvement of multiple independent reviewers and detailed matrices to show how we arrived at the results, adds to reliability of the results. Our study is also in alignment with the United Nation’s sustainable development goal 3.4 [60] by trying to gather evidence on adherence to diabetes self-care using mHealth apps and thereby contributing to reducing diabetes related premature mortality.

We have used narrative synthesis for this integrative review due to the broad variation of the study designs of the included studies. We have attempted to describe the included studies, explore relationships within and between the studies, identify factors influencing the results, and assess the robustness of synthesis [41]. However, the heterogeneity of the included studies has prevented in-depth exploration of relationships between results as suggested in the narrative synthesis [41]. There was a wide variation in the designs and implementation methods used in the studies. The mHealth app interventions varied in terms of duration, type of features offered, app demonstrations given prior to the intervention’s start, and ongoing support. The baseline HbA1C level indicating blood glucose control set for inclusion of participants also varied from not having any set criteria to indication of HbA1C baseline levels of 7–10%. Studies also differed in other baseline inclusion requirements such as current use of a smartphone. Other systematic reviews have shown factors such as duration of intervention [59, 61] and the mean HbA1C level of participants at baseline [59] can significantly impact outcomes. With regard to PROMs, the scales used varied widely (Table 3) within each PROM. Therefore, the results could only be represented as a vote count.

The quality of the ten full-scale studies included in this paper ranges from high to moderate. We have used a percentage calculation similar to a number of other studies to help classify quality [62–64]. A downside of this method, however, is that certain criteria in the appraisal questionnaire may carry more weight than others in deciding overall quality, which is not taken into consideration here. We chose not to classify the four pilot trials included in the study into quality categories as pilot trials assess feasibility using small sample sizes and do not truly fit any of the quality appraisal instruments. In the included studies only two [28, 34] used mHealth apps with a traceable medical device regulation class.

We have limited our inclusion criteria to mHealth apps that have at least one or more of the features like Blood glucose monitoring, Insulin dose, or Insulin bolus calculation. The reason behind this was to look at more diabetes-specific mHealth apps. We also excluded studies conducted before 2010 as the first smartphone with modern-day capabilities was launched in 2007 and the corresponding application store was launched in 2008 [65]. This, along with 4G mobile communication services which were launched in 2010 [66], helped make mHealth apps easily accessible outside the research world [67]. In a 2012 survey study in the US conducted by Pew Research Centre, only two diabetes or blood sugar tracking apps were reported [68]. Considering these developments, we chose to limit the publication dates of included studies to the period 2010 to 2021.

All studies meeting our criteria were from high-income economies [50]. There is a lack of studies looking into type 1 diabetes in upper-middle income, lower-middle income, and low income economies. There could be variation in people’s need for diabetes self-care related mHealth app features as well as factors affecting use based on socioeconomic conditions prevalent in an area. Due to the above stated reasons, our findings cannot be generalised to populations outside high-income economies.

## Conclusion

We found that self-care data monitoring features are the most commonly used features on mHealth apps among people with type 1 diabetes. Personal factors and app design factors were commonly found to affect mHealth app use from perspectives of people with type 1 diabetes. Only two studies reported significant improvement in any of the PROMs used in studies. We are unable to come to a strong conclusive result in any of our three research questions due to the limited number of studies reporting on these aspects among people with type 1 diabetes, as well as the heterogeneity of the studies. We therefore recommend further large-scale studies in these areas focusing on perspectives of people with type 1 diabetes that can ultimately improve mHealth app use for self-care.

## Supplementary Information

**Additional file 1.**

**Additional file 2.**

**Additional file 3.**

## Data Availability

All the data and materials generated during this study are included in this paper or as supplementary files to this paper.

## Abbreviations

ADCES: Association of Diabetes Care and Education Specialists
App: Application
CINAHL: Cumulative Index to Nursing and Allied Health Literature
HbA1C: Hemoglobin A1C or Glycated hemoglobin
IEEE: Institute of Electrical and Electronics Engineers
mHeath apps: Mobile health applications
PROM: Patient reported outcome measures
PROSPERO: The International Prospective Register of Systematic Reviews
WHO ICTRP: World health organization’s International Clinical Trials Registry Platform
4G: 4^Th^ generation
