# Supplementary material for: Using mHealth applications for self-care – An integrative review on perceptions among adults with type 1 diabetes
Source: BMC Endocr Disord. 2022 May 25;22:138. doi: 10.1186/s12902-022-01039-x (PMC9131554; doi:10.1186/s12902-022-01039-x)
Supplement: Supplementary file 1 — Additional file 1. [file 12902_2022_1039_MOESM1_ESM.docx]

**Supplementary Table 1: Tailored Search strategy for each database**

| **Database** | **Search string** | **No: of hits** |
| --- | --- | --- |
| PubMed TiAb* | ("diabetes mellitus"[MeSH Terms] OR ("diabetes"[All Fields] AND "mellitus"[All Fields]) OR "diabetes mellitus"[All Fields] OR "non insulin dependant diabetes mellitus"[All Fields] OR "type 2 diabetes mellitus"[All Fields] OR T2DM[All Fields] OR "insulin dependant diabetes mellitus"[All Fields] OR "type 1 diabetes mellitus"[All Fields] OR T1DM[All Fields]) AND ("blood glucose self-monitoring"[MeSH Terms] OR blood glucose self-monitoring[Title/Abstract] OR "self management"[Title/Abstract] OR "self-management"[MeSH Terms] OR "self care"[Title/Abstract] OR "self care"[MeSH Terms] OR physiologic monitoring[Title/Abstract] OR "self monitor"[Title/Abstract] OR ("self"[All Fields] AND "monitoring, physiologic"[MeSH Terms]) OR "self-assessment"[MeSH Terms] OR "self-assessment"[Title/Abstract] OR "diagnostic self evaluation"[MeSH Terms] OR self evaluation[Title/Abstract]) AND (smartphone[Title/Abstract] OR "smartphone"[MeSH Terms] OR smartphone'[Title/Abstract] OR smartphone's[Title/Abstract] OR smartphones[Title/Abstract] OR smartphones'[Title/Abstract] OR "mobile medical application*"[Title/Abstract] OR telemonitoring[Title/Abstract] OR telemonitor[Title/Abstract] OR "tele-monitor"[Title/Abstract] OR "tele-monitoring"[Title/Abstract] OR telehealth[Title/Abstract] OR "tele-health"[Title/Abstract] OR "ehealth"[Title/Abstract] OR "e-health"[Title/Abstract] OR "telemedicine"[MeSH Terms] OR mhealth[Title/Abstract] OR m-health[Title/Abstract] OR "mobile health"[Title/Abstract] OR telemedicine[Title/Abstract] OR "mobile app"[Title/Abstract] OR "mobile apps"[Title/Abstract] OR "digital health"[Title/Abstract] OR "medical informatics"[Title/Abstract] OR "health informatics"[Title/Abstract] OR "medical informatics"[MeSH Terms] OR "medical informatics application"[Title/Abstract] OR "medical informatics applications"[MeSH Terms] OR "Nursing Informatics"[Mesh] OR "Nursing informatics"[Title/Abstract] OR "Telenursing"[Mesh] OR "telenursing"[Title/Abstract]) | 1445 |
| CINAHL  TiAb* | S1 AND S2 AND S3  **S1** : ( diabetes mellitus OR "non insulin dependant diabetes mellitus" OR "type 2 diabetes mellitus" OR T2DM OR "insulin dependant diabetes mellitus" OR "type 1 diabetes mellitus" OR T1DM ) OR ( (MH "Diabetes Mellitus, Type 2") OR (MH "Diabetes Mellitus, Type 1") OR (MH "Diabetes Mellitus") )  Search modes - Find all my search terms  **S2:** TI ( ( "health informatic*" OR smartphone* OR ( telemonitoring OR telemonitor OR "tele monitor*" OR "tele-monitor*") OR ( telehealth OR tele-health OR "tele health" ) OR ( ehealth OR e-health ) OR ( mHealth OR m-health OR "mobile health" ) OR ( "mobile app*" OR mobile-app* OR"mobile medical application*" ) OR "digital health" OR ( "medical informatics application" OR "medical informatic*" OR "Nursing Informatics" OR "telenursing" ) ) OR ( (MH "Health Informatics") OR (MH "Medical Informatics") OR (MH "Nursing Informatics") ) OR ( (MH "Telemedicine") OR (MH "Telehealth") ) OR (MH "Telenursing") OR (MH "Mobile Applications") OR (MH "Smartphone") ) OR AB ( ( "health informatic*" OR smartphone* OR ( telemonitor* OR "tele monitor*" OR "tele-monitor*" ) OR ( telehealth OR tele-health OR "tele health" ) OR ( ehealth OR e-health ) OR ( mHealth OR m-health OR "mobile health" ) OR ( "mobile app*" OR mobile-app* OR "mobile medical application*" ) OR "digital health" OR ( "medical informatics application" OR "medical informatic*" OR "Nursing Informatics" OR "telenursing") ) OR ( (MH "Health Informatics") OR (MH "Medical Informatics") OR (MH "Nursing Informatics") ) OR ( (MH "Telemedicine") OR (MH "Telehealth") ) OR (MH "Telenursing") OR (MH "Mobile Applications") OR (MH "Smartphone") ) Search modes - Find all my search terms  **S3:** TI ( ( "self manag*" OR "blood glucose self-monitoring" OR "self assess*" OR "self evaluat*" OR "Self monitor*" OR "Self care") OR (MH "Self Assessment") OR ( (MH "Self-Management") OR (MH "Self Care") OR (MH "Blood Glucose Self-Monitoring") ) ) OR AB ( ( "self manag*" OR "blood glucose self-monitoring" OR "self assess*" OR "self evaluat*" OR "Self monitor*" OR "Self care") OR (MH "Self Assessment") OR ( (MH "Self-Management") OR (MH "Self Care") OR (MH "Blood Glucose Self-Monitoring") ) ) Search modes - Find all my search terms | 575 |
| Web of Science  TiAbKey^#^ | TOPIC: (diabetes mellitus OR "non insulin dependant diabetes mellitus" OR "type 2 diabetes mellitus" OR T2DM OR "insulin dependant diabetes mellitus" OR "type 1 diabetes mellitus" OR T1DM) AND TOPIC: ("self manag*" OR "blood glucose self monitoring" OR "self assess*" OR "self care" OR "self monitor*" OR "self evaluat*") AND TOPIC: ("health informatics" OR "nursing informatics" OR telenursing OR "telemonitor*" OR "tele-monitor*" OR telehealth OR "ehealth" OR "e-health" OR "m-health" OR "mobile health" OR mhealth OR "mobile app*" OR "mobile medical application*" OR "digital health" OR "medical informatic*" OR smartphone*)  Databases= WOS, KJD, MEDLINE, RSCI, SCIELO Timespan=All years  Search language=Auto | 1152 |
| SCOPUS  TiAbKey^#^ | ( TITLE-ABS-KEY ( smartphone* OR "health informatics" OR "nursing informatics" OR telenursing OR "telemonitor*" OR "tele-monitor*" OR telehealth OR "ehealth" OR "e-health" OR "m-health" OR "mobile health" OR mhealth OR "mobile app*" OR "mobile medical application*" OR "digital health" OR "medical informatic*" ) ) AND ( TITLE-ABS-KEY ( "diabetes mellitus" OR "non insulin dependant diabetes mellitus" OR "type 2 diabetes mellitus" OR t2dm OR "insulin dependant diabetes mellitus" OR "type 1 diabetes mellitus" OR t1dm ) ) AND ( TITLE-ABS-KEY ( "self manag*" OR "blood glucose self monitoring" OR "self assess*" OR "self care" OR "self monitor*" OR "self evaluat*" ) ) AND ( EXCLUDE ( DOCTYPE , "no" ) OR EXCLUDE ( DOCTYPE , "le" ) OR EXCLUDE ( DOCTYPE , "ed" ) OR EXCLUDE ( DOCTYPE , "ch" ) OR EXCLUDE ( DOCTYPE , "er" ) ) | 1246 |
| PsychInfo TiAb* | (ab(Smartphone* OR "mobile app*" OR "mobile health" OR telemedicine) OR su(Smartphone* OR "mobile app*" OR "mobile health" OR telemedicine) OR TIAB(telemonitor* OR "tele-monitor" OR telehealth OR "tele-health" OR ehealth OR "e-health" OR mHealth OR "m-health" OR "digital health" OR "health informatics" OR "medical informatics" OR "Nursing Informatics" OR Telenursing OR "mobile medical application*")) AND (ab("Self management" OR "Self Care") OR su("Self management" OR "Self Care") OR TIAB("self manag*" OR "blood glucose self monitoring" OR "self assess*" OR "Self care" OR "self monitor*" OR "self evaluat*")) AND (ab(Diabetes Mellitus) OR su(Diabetes Mellitus)) AND PEER(yes) | 115 |
| IEEExplore | ((diabetes mellitus OR "non insulin dependant diabetes mellitus" OR "type 2 diabetes mellitus" OR T2DM OR "insulin dependant diabetes mellitus" OR "type 1 diabetes mellitus" OR T1DM refined by:ContentType:Conferences, ContentType:Journals, ContentType:Early Access Articles; )) AND ("self management" OR "self manage*" OR "blood glucose self monitoring" OR "self care" OR "self monitor*" OR "self evaluat*" OR "Self Assess*" refined by:ContentType:Conferences, ContentType:Journals, ContentType:Early Access Articles; ) | 109 |

*TiAb- title & abstract search, ^#^ TiAbKey- title, abstract & keyword search
