# Supplementary material for: Using mHealth applications for self-care – An integrative review on perceptions among adults with type 1 diabetes
Source: BMC Endocr Disord. 2022 May 25;22:138. doi: 10.1186/s12902-022-01039-x (PMC9131554; doi:10.1186/s12902-022-01039-x)
Supplement: Supplementary file 2 — Additional file 2. [file 12902_2022_1039_MOESM2_ESM.docx]

**Supplementary table S2: Quality appraisal along with ethical approval & conflict of interest**

**Table S2.1. Quality Appraisal according to JBI Checklist for Randomized Controlled Trials along with ethical approval & conflict of interest**

| **Author/ year** | Q1 | Q2 | Q3 | Q4 | Q5 | Q6 | Q7 | Q8 | Q9 | Q10 | Q11 | Q12 | Q13 | % Score | Quality appraisal | Ethics appro-val | Conflict of interest |
| --- | --- | --- | --- | --- | --- | --- | --- | --- | --- | --- | --- | --- | --- | --- | --- | --- | --- |
| Di Bartolo, P., et al. (2017) | Y | Y | Y | N | N | U | Y | Y | Y | Y | Y | Y | Y | 76.9 | High | Yes | No |
| Drion, I., et al. (2015) | Y | Y | Y | N | U | U | Y | Y | Y | Y | Y | Y | Y | 76.9 | High | Yes | No |
| Kirwan, M., et al. (2013) | Y | U | N | N | N | U | U | Y | Y | Y | Y | Y | Y | 53.84 | Moderate | Yes | No |

Y= yes; N= no; U= unclear; N/A= not applicable,? = unsure

Q1. Was true randomization used for assignment of participants to treatment groups?

Q2. Was allocation to treatment groups concealed?

Q3. Were treatment groups similar at the baseline?

Q4. Were participants blind to treatment assignment?

Q5. Were those delivering treatment blind to treatment assignment?

Q6. Were outcomes assessors blind to treatment assignment?

Q7. Were treatment groups treated identically other than the intervention of interest?

Q8. Was follow up complete and if not, were differences between groups in terms of their follow up adequately described and analyzed?

Q9. Were participants analyzed in the groups to which they were randomized?

Q10. Were outcomes measured in the same way for treatment groups?

Q11. Were outcomes measured in a reliable way?

Q12. Was appropriate statistical analysis used?

Q13. Was the trial design appropriate, and any deviations from the standard RCT design (individual randomization, parallel groups) accounted for in the conduct and analysis of the trial?

**Table S2.2. Quality Appraisal according to JBI Checklist for Quasi-Experimental Studies (non-randomized experimental studies) along with ethical approval & conflict of interest**

| **Author/year** | Q1 | Q2 | Q3 | Q4 | Q5 | Q6 | Q7 | Q8 | Q9 | % score | Quality appraisal | Ethics approval | Conflict of interest |
| --- | --- | --- | --- | --- | --- | --- | --- | --- | --- | --- | --- | --- | --- |
| Feuerstein-Simon, C., et al. (2018) | Y | N | U | N | Y | U | Y | Y | U | 44.44 | Pilot study | Yes | No |
| Jeon, E., & Park, H. A. (2019) | Y | Y | U | N | Y | Y | Y | Y | Y | 77.78 | Pilot study | Yes | No |
| Mora, P., et al. (2017) | Y | Y | U | N | Y | N | Y | Y | Y | 66.67 | Pilot study | Yes | ? |
| Skrøvseth, S. O., (2012) | Y | Y | U | N | Y | Y | Y | Y | Y | 77.78 | High | NR | No |
| Tack, C., et al. (2018) | Y | Y | N | N | Y | Y | Y | Y | Y | 77.78 | High | Yes | No |
| Årsand, E., et al. (2015) | Y | U | U | N | U | Y | N | U | U | 22.22 | Pilot study | NR | No |

Y= yes; N= no; U= unclear; N/A= not applicable; NR: not reported; ?= unsure

Q1. Is it clear in the study what is the ‘cause’ and what is the ‘effect’ (i.e. there is no confusion about which variable comes first)?

Q2. Were the participants included in any comparisons similar?

Q3. Were the participants included in any comparisons receiving similar treatment/care, other than the exposure or intervention of interest?

Q4. Was there a control group?

Q5. Were there multiple measurements of the outcome both pre and post the intervention/exposure?

Q6. Was follow up complete and if not, were differences between groups in terms of their follow up adequately described and analyzed?

Q7. Were the outcomes of participants included in any comparisons measured in the same way?

Q8. Were outcomes measured in a reliable way?

Q9. Was appropriate statistical analysis used?

**Table S2.3. Quality Appraisal according to JBI Checklist for Cross Sectional Studies along with ethical approval & conflict of interest**

| **Author/year** | Q1 | Q2 | Q3 | Q4 | Q5 | Q6 | Q7 | Q8 | % score | Quality Appraisal | Ethics approval | Conflict of interest |
| --- | --- | --- | --- | --- | --- | --- | --- | --- | --- | --- | --- | --- |
| Boyle, L., et al. (2017) | Y | Y | Y | Y | N | N | U | Y | 62.5 | Moderate | NR | No |
| Trawley, S., et al. (2017) | Y | Y | Y | Y | U | N/A | Y | Y | 85.7 | High | Yes | No |
| Zahed, K., et al. (2020) | Y | Y | Y | U | Y | Y | U | Y | 75 | High | Yes | No |

Y= yes; N= no; U= unclear; N/A= not applicable; ?= unsure, NR= not reported

Q1. Were the criteria for inclusion in the sample clearly defined?

Q2. Were the study subjects and the setting described in detail?

Q3. Was the exposure measured in a valid and reliable way?

Q4. Were objective, standard criteria used for measurement of the condition?

Q5. Were confounding factors identified?

Q6. Were strategies to deal with confounding factors stated?

Q7. Were the outcomes measured in a valid and reliable way?

Q8. Was appropriate statistical analysis used?

**Table S2.4. Quality Appraisal according to JBI Checklist for Qualitative Research design along with conflict of interest**

| **Author/year** | Q1 | Q2 | Q3 | Q4 | Q5 | Q6 | Q7 | Q8 | Q9 | Q10 | % score | Quality Appraisal | Conflict of interest |
| --- | --- | --- | --- | --- | --- | --- | --- | --- | --- | --- | --- | --- | --- |
| Knight, B. A., et al. (2016) | U | Y | Y | Y | Y | N | N | Y | Y | Y | 70 | High | No |
| Ritholz, M. D., et al. (2019) | Y | Y | Y | Y | Y | Y | U | Y | Y | Y | 90 | High | ? |

Y= yes; N= no; U= unclear; N/A= not applicable, ?= unsure

* Ethical approval column not included as it is part of Q9

Q1. Is there congruity between the stated philosophical perspective and the research methodology?

Q2. Is there congruity between the research methodology and the research question or objectives?

Q3. Is there congruity between the research methodology and the methods used to collect data?

Q4. Is there congruity between the research methodology and the representation and analysis of data?

Q5. Is there congruity between the research methodology and the interpretation of results?

Q6. Is there a statement locating the researcher culturally or theoretically?

Q7. Is the influence of the researcher on the research, and vice- versa, addressed?

Q8. Are participants, and their voices, adequately represented?

Q9. Is the research ethical according to current criteria or, for recent studies, and is there evidence of ethical approval by an appropriate body?

Q10. Do the conclusions drawn in the research report flow from the analysis, or interpretation, of the data.
