# Supplementary material for: Using mHealth applications for self-care – An integrative review on perceptions among adults with type 1 diabetes
Source: BMC Endocr Disord. 2022 May 25;22:138. doi: 10.1186/s12902-022-01039-x (PMC9131554; doi:10.1186/s12902-022-01039-x)
Supplement: Supplementary file 3 — Additional file 3. [file 12902_2022_1039_MOESM3_ESM.docx]

**Table S3: mHealth application features, which help, promote and maintain diabetes self-care**

| **Study** | **Data entry** | **App Display** | **Self-care data monitoring** | | | | **Feedback & reminders** | **Data sharing** | **Others** |
| --- | --- | --- | --- | --- | --- | --- | --- | --- | --- |
|  |  |  | **Blood glucose** | **Insulin log & bolus calculator** | **Diet** | **Physical activity** |  |  |  |
| Boyle, L., et al. (2017) | - | - | Blood glucose diaries. | Calculation device for insulin dose. | Diary of meals and carbohydrate intake. | - | Reminders to check blood sugar. | - | - |
| Knight, B. A., et al. (2016) | Automatic data transmission to the app.  Retrospective data editing option.  Free text options to record pertinent events and predetermined list of events. | Personalized screen displays allowing removal (or cloaking) of features- simplifies user interface. | - | Customizable time settings for insulin algorithms,  Reverse (carbohydrate) calculator- to prevent/manage hypoglycemia and the insulin bolus adjustment feature for alcohol intake. | Food database connectivity.  A shortcut menu to ‘register’ favorite foods. | - | - | Additional web based storage function. | - |
| Ritholz, M. D., et al. (2019) | - | Graphs provided led to less worries about hypo- or hyperglycemia. | - | - | - | - | Constant and immediate feedback- increase knowledge & management of glucose variability. | - | - |
| Tack, C., et al. (2018) | Wireless/ Bluetooth connections, for automated data entry between their blood glucose meter or insulin pump and the app. | The graphic display of blood glucose (trend). | - | A more advanced insulin bolus suggestions specific for different activities like sports. | - | - | - | Data sharing options, such as easy exporting of data, use of cloud solutions, or a connection with their personal health record. | - |
| Trawley, S., et al. (2017) | - | - | Recording blood glucose levels. | - | Carbohydrate counting feature. | Tracking physical activity. | - | - | - |
| Zahed, K., et al. (2020) | - | Graphical display of data. | Glucose monitoring and log for abnormal sugar levels. | Insulin log. | Food log. | - | Reminders. | - | Medication log and Educational content. |
| Årsand, E., et al. (2015) | Order of data entry as blood glucose, insulin, followed by carbs  Make new registrations quickly  Registrations of events (illness, stress, etc.). | Ability to see last measurement values.  Able to view blood glucose statistics/ graph on the smartwatch. | - | Be able to label different insulin types. | Add notes for carbohydrates & enter more exact carbohydrate levels. | Automatic import of physical activities. | A blood glucose reminder 90 minutes after a meal. | - | - |
